# A history of experimental phasing in macromolecular crystallography

**DOI:** 10.1107/S205979831501476X

**Published:** 2016-03-01

**Authors:** Neil Isaacs

**Affiliations:** a Department of Chemistry, University of Glasgow, Glasgow G12 8QQ, Scotland

**Keywords:** history, experimental phasing

## Abstract

It was just over a century ago that W. L. Bragg published a paper describing the first crystal structures to be determined using X-ray diffraction data. These structures were obtained from considerations of X-ray diffraction (Bragg equation), crystallography (crystal lattices and symmetry) and the scattering power of different atoms. Although W. H. Bragg proposed soon afterwards, in 1915, that the periodic electron density in crystals could be analysed using Fourier transforms, it took some decades before experimental phasing methods were developed. Many scientists contributed to this development and this paper presents the author’s own perspective on this history. There will be other perspectives, so what follows is *a* history, rather than *the* history, of experimental phasing.

## Abstract 2

The development of phasing methods in protein crystallography is presented.

## 1. Introduction   

According to von Laue (1969 ▸), crystallography, defined as the study of crystals, began in 1611 with the publication by the great astronomer Johannes Kepler of a small pamphlet on hexagonal snow. Three hundred years later, when Friedrich, Knipping and Laue first observed the diffraction of X-rays by a crystal, the physical and mathematical study of crystals had established the concepts of crystal lattices, Miller indices, unit cells, crystal symmetry and space groups [readers are encouraged to read Kubbinga (2012 ▸) for a description of the history of these developments]. It was against this background that W. L. Bragg set out to explain Laue’s result in terms of the crystal structure.

## 2. The first structures   

Bragg’s great insight was to realise that the diffraction effect recorded by Laue could be considered as a reflection of X-rays from lattice planes of the crystal. This led to the formulation of the Bragg equation and, by considering the distribution of scattering centres in face-centred cubic lattices, to the structures of zincblende (ZnS) and simple alkaline halides (Bragg, 1913 ▸). This type of ‘trial-and-error’ approach, where diffraction data calculated from a proposed structure are compared with the observed experimental data, does not require any prior knowledge of phases and became the norm for the determination of crystal structures for decades.

## 3. Fourier methods   

It was only two years after W. L. Bragg’s 1913 paper that his father discussed the possibility of using Fourier’s methods to analyse the periodic variation of density in a crystal (Bragg, 1915 ▸). However, these ideas were not pursued for another decade until William Duane, who had been appointed to a chair of Biophysics at Harvard (and was probably the first Professor of Biophysics in the world), used quantum theory to derive an expression to calculate the diffracting power (electron density) at points in a crystal using Fourier transform notation (Duane, 1925 ▸). These equations included phase angles explicitly and possibly mark the first allusion to phasing in the crystallographic literature. These ideas were used by a graduate student, Robert Havighurst, to calculate the electron density in NaCl crystals (Havighurst, 1925 ▸). The form of the equation he gives for the electron density at points in a centric space group is one that is now very familiar to all X-ray crystallographers. These ideas were developed further by W. L. Bragg (Bragg, 1929 ▸). In centric space groups the phases are either 0 or 180°, giving structure-factor signs of ±1. Using the known structure of the silicate diopside [CaMg(SiO_3_)_2_], Bragg discussed how electron-density maps could be calculated in projection for crystals with centres of symmetry and showed how they could be used to estimate the electron count of each atom and to improve the accuracy of the coordinates. Furthermore, he showed how knowledge of the positions of the heavy atoms was sufficient to fix the signs of all structure factors, allowing the positions of the lighter O atoms to be determined from the Fourier map. While this became the standard procedure used by X-ray crystallographers for many years, its use was restricted for some years to centric crystals, where the location of the heavy atom(s) could be determined from considerations of symmetry or by trial and error.

## 4. Patterson methods   

A breakthrough came in 1934 when Arthur Lindo Patterson calculated Fourier maps using *F* ^2^ (which have no phases) as coefficients (Patterson, 1934 ▸). However, as reflected in the title of his paper, *A Fourier series method for the determination of the components of interatomic distances in crystals*, this was not immediately seen as a method for determining the location of a heavy atom. It was David Harker, working in Linus Pauling’s laboratory, who showed that space-group symmetry could restrict the location of interatomic vectors to certain lines or sections of the Patterson map and that a reordering of the form of the calculation for these lines and sections would allow all of the diffraction data to be used with a manageable amount of manual computation (Harker, 1936 ▸). This was important as using all of the three-dimensional data removed the possibility of overlapping peaks that could occur when only line (*e.g.* *h*00) or projection (*e.g.* *h*0*l*) data were used. The combination of Patterson methods with heavy-atom phasing proved to be very successful in determining structures of small molecules substituted with heavy atoms such as the halides. Using these procedures, Cox & Jeffrey (1939 ▸) determined the isomorphous structures of glucosamine hydrobromide and hydrochloride, which crystallize in space group *P*2_1_. This was probably the first noncentric structure to be determined in this way. However, these methods did not work with proteins. The large number of atoms in proteins meant that the heavy-atom vectors often could not be recognized against the crowded background of small-atom vectors, and even when the heavy atoms could be located the phases calculated were insufficiently accurate to provide an interpretable electron-density map.

## 5. Isomorphous replacement   

Isomorphous replacement was used by Cork (1927 ▸) in a series of alum structures and later by Robertson (1937 ▸) in his study of free and metal-substituted phthalocyanine structures. In both of these cases the metal was located on a centre of symmetry, so that a comparison of the change in magnitude of *F* on substitution (or removal, in the case of phthalocyanine) of the metal allowed determination of its sign. In their paper on the structures of glucosamine hydrobromide and hydrochloride, Cox & Jeffrey (1939 ▸) state that phase angles were obtained by comparison of corresponding *F* values for chloride and bromide. As they provided no further details, it is unclear whether this could be an early instance of phasing by isomorphous replacement. No further applications seem to have taken place until 1951, when Bijvoet and coworkers reported in some detail how sulfate and selenite forms of strychnine were used to estimate phases by the method of isomorphous substitution (Bokhoven *et al.*, 1951 ▸). They described how *F* values for noncentric reflections have to be considered as vectors and illustrated with circles and vector triangles how phase estimates could be obtained, but with an ambiguity in sign. Using structure factors with both phase estimates to calculate a double Fourier gave a map displaying both the true structure and its mirror image. A projection onto a centric plane showed a clear benzene ring and provided a starting point for a separation of the two images using known interatomic distances and valence angles. In this same paper they described how double isomorphous replacement could resolve the phase ambiguity, but they did not proceed with this.

The first application to proteins came from Perutz, who used a single isomorphous replacement (Hg derivative) to phase centric reflections for horse haemoglobin data (Green *et al.*, 1954 ▸). In the same year Bijvoet discussed how anomalous scattering could be used to overcome the phase ambiguity that arises from single isomorphous replacement (Bijvoet, 1954 ▸). The method could not be applied at the time owing to the inability of the available instrumentation to record the small differences in scattering with sufficient accuracy, and it would take another seven years before the method was revisited (Blow & Rossmann, 1961 ▸). Meanwhile, in a comprehensive paper published in 1956, David Harker described in great detail how phases could be obtained from double isomorphous replacement data and how to overcome the problems of origin choice and enantiomorphism (Harker, 1956 ▸). He also discussed the problem of non-isomorphism owing, for example, to small changes in unit-cell dimensions and gave a rule of thumb linking the fractional changes in unit-cell dimensions to the resolution limit of useful data. Later that year, Perutz addressed the problems of locating the position of the heavy atoms and of finding their correct relative locations in different compounds (Perutz, 1956 ▸), while Crick & Magdoff (1956 ▸) gave estimates of the average change in intensity owing to adding a heavy atom to a protein crystal. They also gave formulae for the changes owing to small translations and rotations of the molecules, alterations of the unit-cell parameters and by ‘breathing’ movements. They showed that small molecular shifts would affect the isomorphous replacement method at high resolutions, but not at low resolutions. As a consequence of these factors contributing to non-isomorphism, together with errors in measuring structure factors and scaling different sets of data together, phases estimated by isomorphous replacement had considerable errors. Blow & Crick (1959 ▸) addressed this problem and derived expressions for the ‘best’ Fourier (the Fourier transform expected to have the minimum mean-square difference from the Fourier transform of true *F* values) and a weighting scheme based on estimates of the correctness of the phase (figure-of-merit weighting). With the publication of this paper, it could be argued that the isomorphous replacement method of phasing was now firmly established, although many other papers published subsequently dealt with associated issues such as the use of anomalous scattering with single isomorphous replacement (Blow & Rossmann, 1961 ▸; North, 1965 ▸, Matthews, 1966 ▸) and the effects of phase bias and reliability of derivatives (Dickerson *et al.*, 1967 ▸).

## 6. Molecular replacement   

In 1955, in a study of reduced human haemoglobin using Patterson projection maps, Perutz found a resemblance to corresponding maps from horse methaemoglobin and inferred that the two proteins shared a similarity in structure (Perutz *et al.*, 1955 ▸). This study was carried out by visual inspection of low-resolution projection maps. In the early 1960s, with growing evidence that proteins like myoglobin and haemoglobin could contain subunits related by noncrystallographic symmetry, Rossmann and Blow developed a method for detecting this partial, approximate symmetry using only intensity data (Rossmann & Blow, 1962 ▸). Although the title of their paper referred only to *The Detection of Sub-Units Within the Crystallographic Asymmetric Unit*, they did anticipate that ‘this ‘redundancy’ in information might be used to help solve a structure’ and that ‘…the ideas presented here are as applicable to finding the relationship between similar molecules in different crystal lattices’. Molecular replacement is now the major procedure used to phase protein structures.

## 7. Anomalous scattering   

Anomalous scattering measurements were initially used to overcome the sign ambiguity in phases obtained from single isomorphous replacement experiments. As diffraction data were collected at a single wavelength, the strength of the anomalous scattering depended on the nature of the introduced heavy atom. Anomalous difference data could also be used in Patterson maps to position the anomalous scatterer. Hendrickson and Teeter showed, with the direct determination of the structure of crambin, that the small anomalous scattering signal from intrinsic S atoms was sufficient to phase the data (Hendrickson & Teeter, 1981 ▸). At the same time, the introduction of tuneable synchrotron sources made possible the collection of anomalous data at a number of wavelengths (Phillips *et al.*, 1977 ▸) and the direct determination of structures from multiple-wavelength anomalous dispersion (MAD) phasing, as exemplified by the structure of a basic blue copper protein (Guss *et al.*, 1988 ▸). With the development of selenomethionyl proteins by Hendrickson (Yang *et al.*, 1990 ▸), anomalous scattering has become the first method of choice for phasing new protein structures.
